# The comparison of anterior knee pain in severe and non severe arthritis of the lateral facet of the patella following a mobile bearing unicompartmental knee arthroplasty

**DOI:** 10.1186/s40064-016-1914-1

**Published:** 2016-02-27

**Authors:** Boonchana Pongcharoen, Chaivet Reutiwarangkoon

**Affiliations:** Department of Orthopaedic Surgery, Faculty of Medicine, Thammasat University, Pathumthani, Thailand

**Keywords:** Oxford, Unicompartmental knee arthroplasty, Patellofemoral arthritis, Pain, Functional score

## Abstract

In the past, medial osteoarthritis (OA) knee with symptomatic patellofemoral (PF) arthritis has not been recommended for a unicompartmental knee arthroplasty (UKA). However, recent studies have reported that UKA has shown good results in patients with medial OA of the knee, including those with PF arthritis. The purpose of this study is to compare the results between patients with medial OA knees; those with severe arthritis of the lateral facet of the patella and patients without severe arthritis of the lateral facet of the patella following mobile bearing UKA. We have prospectively evaluated 104 patients (114 knees) who had undergone an Oxford mobile bearing UKA. The mean follow-up was 19.05 months (range 12.30–29.70 months). The patients were divided into two groups: group I consisted of eighty patients (88 knees) who did not have severe arthritis of the lateral facet (Outerbridge grade 0–2) and group II had twenty-four patients (26 knees) who had severe arthritis of the lateral facet (Outerbridge grade 3, 4). We recorded the incidence of anterior knee pain, knee scores, pain scores, and functional scores in comparison of the two groups. The visual analog scale (VAS) and incidence of post-operative anterior knee pain had not shown any significant differences. The VAS for post-operative anterior knee pain was 0.11 (SD 0.56, range 0–3 point) versus 0.12 (SD 0.59, range 0–3 point) for group I and group II patients, respectively (*P* = 0.98). The incidence of post-operative anterior knee pain was 4.5 versus 3.8 % for group I and group II patients, respectively (*P* = 0.88). The pain scores and functional scores had not exhibited any differences. However, the knee scores of patients with severe arthritis of the lateral facet of the patella was worse than those seen in patients without severe arthritis of the lateral facet of the patella with a statistical significance. It was scored as 96.78 (SD 4.56, range 85–100) versus 94.43 (SD 4.50, range 81–100) for group I and group II patients, respectively (*P* = 0.02). Anterior knee pain, pain scores, and functional scores were not different between the two groups following a medial Oxford UKA. However, the knee scores of patients with severe arthritis of the lateral facet were worse than those in patients without severe arthritis of the lateral facet of the patella.

## Background

Medial unicompartmental knee arthroplasty (UKA) has shown good long-term results (Murray et al. 1998; Berger et al. 1999), high patient satisfaction (Mercier et al. 2010), a high rate of return to sport activities (Pietschmann et al. 2013), and simplified procedures during conversion to total knee arthroplasty (TKA) (Saragaglia et al. 2009). UKA has also shown lower blood loss (Jeer et al. 2005; Pongcharoen and Reutiwarangkoon 2016), lower morbidity rates (Jeer et al. 2005; Pongcharoen and Reutiwarangkoon 2016), and earlier recovery times (Cameon and Jung 1988; Jahromi et al. 2004) than those seen in TKA.

Medial osteoarthritic (OA) knees with symptomatic patellofemoral (PF) arthritis were not recommended for UKA in the past (Kozinn and Scott 1989; Berger et al. 2004). However, the Oxford UKA has shown good clinical results in patients with medial OA knee with symptomatic PF arthritis (Beard et al. 2007a, b; Kang et al. 2011). But, the patient with very severe arthritis of the lateral facet of the patella that include bone loss, grooving, and ebernation still were not recommended for the procedure (Beard et al. 2007a, b). Presently, arthritis of the patellofemoral compartment, especially arthritis of the lateral facet of the patella, is a controversial issue for the recommendation of UKA (Kang et al. 2011; Beard et al. 2007a, b; Song et al. 2016). Therefore, some surgeons choose patients with asymptomatic PF arthritis for UKA (Kozinn and Scott 1989; Berger et al. 2004; Hernigou and Deschamps 2002). Some orthopedists accept symptomatic PF arthritis, but the arthritis has to involve only the medial facet or with minimal involvement of the lateral facet (Song et al. 2016). Some orthopedists ignore the symptom of PF arthritis. However, if the patella has very severe arthritis of the lateral facet with bone loss, grooving and ebernation; total knee arthroplasty (TKA) is recommended as the first line (Beard et al. 2007a, b).

The purpose of this study is to compare the incidence of anterior knee pain and the VAS for anterior knee pain, to determine pain scores, knee scores, and functional scores between patients with medial OA of the knee without severe arthritis of the lateral facet of the patella and patients with severe arthritis of the lateral facet of the patella.

## Methods

A prospective cohort study was conducted between January, 2011 and April, 2013. The Human Research Ethics Committee of faculty of Medicine, Thammasat University has approved the protocol (Reg. no: MTU-EC-OT-0-091/57). The patients who have undergone a medial Oxford UKA (Biomet, Bridgend, UK), performed by a single surgeon (BP), were included in this study. The inclusion criteria were patients who were diagnosed with medial osteoarthritis (OA) of the knee with an Alhback score of 2, 3 and 4 (Ahlback 1968) and with any VAS score for pre-operative anterior knee pain, who were older than 40 years of age, with a ROM greater than 90°, a varus deformity of less than 25°, and a flexion contracture less than 20°. The exclusion criteria were any patients with a diagnosis of spontaneous osteonecrosis of the knee (SPONK), intraoperative ACL insufficiency, inflammatory joints, gout, post-traumatic arthritis, and primary PF arthritis. The patients were divided into two groups based on the intraoperative findings of the lateral facet of the patella by the surgeon (BP) who had used a method by Outerbridge (Outerbridge 1961). The intra-operative finding of cartilage loss of lateral facet of patella was classified into 5 grades; Grade 0 was normal cartilage, grade1 was softening and swelling of articular cartilage, grade 2 was a partial-thickness cartilage loss. The fragmentation and fissuring in an area less than half an inch in diameter, grade 3 was the same as grade 2 but the fragmentation and fissuring in an area more than half an inch in diameter and grade 4 was exposed subchondral bone. Group I had non severe arthritis of the lateral facet of the patella (Outerbridge grade 0–2). Group II had severe arthritis of the lateral facet of the patella (Outerbridge grade 3, 4). The preoperative patients’ demographics include; age, gender, ratio of the site, rage of motion, BMI, the tibiofemoral angle, flexion contractures, recurvatum deformity, incidence of pre-operative anterior knee pain, and VAS for pre-operative anterior knee pain were recorded (Table 1). The mean follow-up was 19.05 months (range 12.30–29.70 months).

**Table 1:** Demographic data

Variable	Non severe arthritis of the lateral facet of the patella (n = 88)	Severe arthritis of the lateral facet of the patella (n = 26)	P value
Age (years)^a^	65.1 ± 6.9 (53–88)	66.0 ± 9.5 (44–84)	0.59
Sex (male/female)	13/75	2/24	0.05
Site (right/left)	43/45	7/19	0.35
BMI (kg/m^2^)^a^	26.3 ± 4.1 (20.0–42.2)	27.5 ± 4.0 (21.4–41.6)	0.19
The Knee Society score© (points)^a^	34.9 ± 2.3 (27–40)	33.8 ± 3.3 (27–44)	0.04
Pain score (points)^a^	12.16 ± 5.1 (0–20)	12.31 ± 7.1 (0–20)	0.91
Functional score (points)^a^	47.22 ± 6.5 (35–65)	44.00 ± 6.1 (30–50)	0.03
ROM (°)^a^	121.0 ± 7.1 (90–130)	112.3 ± 12.3 (90–130)	<0.001
Pre-operative tibiofemoral angle (°)^a^	Varus 3.2 ± 2.6 (0–13)	Varus 9.0 ± 3.9 (5–20)	<0.001
Flexion contracture (°)^a^	4.9 ± 4.2 (1–15)	7.4 ± 5.8 (1–18)	0.02
Recurvatum (°)^a^	1.2 ± 2.8 (5–10)	0.7 ± 2.1 (5–8)	0.45
Anterior knee pain (%)	75 %	84.6 %	0.35
VAS for Anterior knee pain (points)^a^	2.1 ± 1.9 (0–8)	3.2 ± 2.0 (0–6)	0.02

^a^Values are expressed as a mean ± SD, with ranges in parentheses

All surgical procedures were performed by the same surgeon with the Oxford Phase III UKA (Biomet, Bridgend, UK). All patients had received a spinal block with morphine, 0.1–0.2 mg. The patients were then given 1 g of Cefazolin intravenously. A tourniquet was inflated to 300 mm Hg in all cases prior to the skin incision. The anteromedial skin incision was performed from the upper pole of the patella to the medial aspect of the tibial tubercle. A mini-midvastus approach was used in all cases for the prevention of patellar maltracking and the possible resulting, subsequent anterior knee pain (Pongcharoen et al. 2013). The patella was slightly subluxated laterally but was not everted. The osteophytes at the anterior portion of the tibial plateau and the medial condyle of the femur were removed. The tibia was first cut perpendicular to the mechanical axis. The femur was then cut using a femoral intramedullary guide. The femoral cutting guide was set to restore the posterior offset, in the center of the femoral condyle, with 7° of the valgus to the femoral intramedullary guide, and parallel to the tibial axis. The osteophytes of the patella and lateral femoral condyle were removed after finishing the cutting of the tibia and femur. It was easier to remove osteophytes in this step, the knee was extended and the patella rotated 90° for the removal of the osteophytes around the patella. The patella was subluxated laterally for the removal of the osteophytes from the lateral femoral condyle. The severity of the cartilage damage of the patella was recorded at this step. Minimally invasive instrumentation was used in all cases. We used the same instruments to finish the tibia and femur. Thirty millimeters of bupivacaine was injected prior to closing. One intra-articular drain (10-gauge) was inserted prior to suturing the incision. The operative time was recorded.

None of the patients had received antithrombolytic agents. All patients were asked to perform ankle pumps and allowed to walk with partial weight bearing as tolerated at post-operative day 1 for the prevention of deep vein thrombosis and pulmonary embolisms. The patients in both groups also performed quadriceps exercises three times per day for approximately 20 repetitions as soon as possible to improve patellar tracking and reduce anterior knee pain. The Redivac drain™ (Atrium Medical. Hudson, NH, USA) was removed at 24 h post op and blood loss from the drain was recorded. Both groups of patients had received the same discharge instructions and rehabilitation protocols.

We followed patients at 6 weeks, 3 months, 6 months, 1 year, and 2 years post operatively. At each visit, the anterior knee pain was assessed by use of a visual analog scale. Our research assistant asked the patients while performing low chair rising activities and stair climbing. If the patient presented with anterior knee pain, the assistant had them score the pain using a 10-point VAS with 0 representing no pain and 10 representing maximum pain. The number of patients who had anterior knee pain was also recorded. The Knee Society Score© (KSS) (knee score, pain scores, and functional scores) was assessed (Insall et al. 1989). The knee score, pain scores, and functional scores were evaluated by a blinded research assistant at each visit. At each follow-up, we also obtained AP, lateral, skyline view, and long-leg radiographs and recorded the component alignment, tibiofemoral angle, and the joint space width of the medial and lateral facets of the patella. We also recorded patellofemoral complications such as patellar crepitation, and fractures. Patellofemoral complications were assessed by history, physical examination, and imaging. The patients were assessed clinically for DVT by signs and symptoms such as; swelling of the leg, pain in the leg, redness, and increased warmth by history and examination. No DVT screening tests were performed. If the patients were suspected clinically for DVT, they were examined with the use of ultrasonography. All patients who had been diagnosed with DVT were treated by internal medicine physicians. The other complications of infections, loosening of implants, fractures, lateral compartment arthritis, and dislocations of the polyethylene were also recorded.

### Statistics

We have performed a sample-size estimation based on the incidence of anterior pain experienced with the UKA which has shown that 25 patients from each group would be required to exhibit a difference with a risk difference of 20 % (5 vs 25 %) at a significant level of 0.05 and power of 80 %. The VAS for anterior knee pain, pain scores, functional scores, KSS, ROM, knee alignment, tibial slope, operative time, blood loss, BMI, femoral and tibial component alignment were calculated using the Independent Student’s *t* test. The incidence of anterior knee pain, gender, percentage of the type of pain in pain scores, and percentage of the type of function based on functional scores were assessed using the Chi-square test. All 114 knees were included in the data analysis using the SPSS^®^ version 13 (SPSS Inc, Chicago, IL, USA).

## Results

A total of 105 patients (115 knees) were recruited for this study. One patient did not complete the study due to a medial tibial plateau fracture suffered at 3 months postoperatively. This patient had undergone a revision TKA and had shown good results after the surgery. Therefore, a total of 104 patients (114 knees) were included in the final analysis. Group I consisted of 80 patients (88 knees) who had non severe arthritis of the lateral facet of the patella (Outerbridge grade 0–2). Group II consisted of 24 patients (26 knees) who had severe arthritis of the lateral facet of the patellar (Outerbridge 3, 4).

The preoperative patients’ demographics have shown that the patients in group II had a lower range of motion, a greater tibiofemoral angle, a greater ratio of females to males, and a higher VAS for pre-operative anterior knee pain than the patients in group I with a statistical significance. However, there were no differences in age, ratios of site, BMI, flexion contractures, recurvatum deformities, and incidence of pre-operative anterior knee pain (Table 1).

The incidence anterior knee pain and the VAS for post-operative anterior knee pain were not significantly different between the two post-operative groups. The incidence of post-operative anterior knee pain was 4.5 versus 3.8 % in group I and group II patients, respectively (P = 0.88). The VAS for post-operative anterior knee pain was 0.11 (SD 0.56, II range 0–3 point) versus 0.12 (SD 0.59, range 0–3 point) in group I and group II patients, respectively (P = 0.98) (Table 2).

**Table 2:** VAS and occurrence for post operative anterior knee pain

Anterior knee pain (AKP)	Group I (n = 88)	Group II (n = 26)	P value
VAS for post-operative AKP^a^	0.11 ± 0.56 (0–3)	0.12 ± 0.59 (0–3)	0.98
Incidence of post-operative AKP	4.5 % (4/88)	3.8 % (1/26)	0.88

*AKP* anterior knee pain

^a^Values are expressed as mean ± SD, with range in parentheses

The knee scores were with statistically significant differences. It was 96.78 (SD 4.56, range 85–100) versus 94.43 (SD 4.50, range 81–100) in group I and group II patients, respectively (P = 0.02). However the pain scores and functional scores were not different between the two groups (Table 3).

**Table 3:** Post-operative Knee Society score, pain scores, and functional scores

Clinical knee society score	Group I (n = 88)	Group II (n = 26)	P
Knee Society score^a^ (points)	96.78 ± 4.56 (85–100)	94.43 ± 4.50 (81–100)	0.02
Pain score^a^ (point)	48.92 ± 2.57 (40–50)	49.04 ± 2.83 (40–50)	0.84
Functional score^a^ (point)	83.75 ± 5.74 (80–100)	84.04 ± 6.33 (65–90)	0.83

^a^Values are expressed as mean ± SD, with range in parentheses

The radiographic outcome of the patellofemoral compartment was assessed from a skyline radiographic view. Of the 88 pre-operative skyline views of the patients in group I; six knees (6.8 %) were normal, seventy-two knees (81.8 %) had shown medial patellar joint space narrowing, six knees (6.8 %) had narrowing of the medial and lateral facets, and four knees (4.6 %) had an obliteration of the medial patellar joint space. Of the 26 pre-operative skyline views of the group II patients; nineteen knees (73.1 %) had shown a narrowing of the medial and lateral facets, two knees (7.7 %) had an obliteration of the medial facet, four knees (15.4 %) had exhibited an obliteration of the lateral facet, and one knee (3.8 %) had obliteration of the medial and lateral facets.

The post-operative skyline views at the 2 week follow up of the group I patients; seventy-nine knees (89.8 %) were normal, and nine knees (10.2 %) were with medial patellar joint space narrowing. The post-operative skyline view at 2 weeks follow up of the group II patients; eighteen knees (69.2 %) were normal, eight knees (30.8 %) had shown a narrowing of the medial and lateral facets. No patients in either group have developed progressive narrowing of the medial and lateral facets of the patella at the final follow-up.

The blood loss measured from the drains, operative time, and the alignment of the prosthesis were not different between the two groups (Table 4). The postoperative tibiofemoral angle and postoperative ROM were significantly different between the groups as is shown in Table 4. However the post-operative tibiofemoral angles of the two groups were back to a normal alignment.

**Table 4:** Post-operative ROM, component alignment, post-operative tibiofemoral angle, blood loss, and operative time

Category	Group I (n = 88)	Group II (n = 26)	P
Post operative ROM (°)^a^	128.92 ± 5.79 (110–145)	117.69 ± 14.02 (90–130)	<0.001
Post operative tibiofemoral angle (°)^a^	6.10 ± 1.35 (1–10)	4.81 ± 1.96 (2–6)	0.003
Femoral alignment (°)^a^	5.88 ± 1.57 (2–10)	4.88 ± 1.84 (1–9)	0.02
Tibia alignment (°)^a^	0.76 ± 1.02 (2° of valgus–3° of varus)	0.96 ± 1.31 (2° of valgus–3° of varus)	0.41
Posterior slope of tibia (°)^a^	6.76 ± 1.86 (2–10)	6.57 ± 2.12 (2–10)	0.67
Blood loss from drain (ml)^a^	140.29 ± 82.04 (10–400)	125.79 ± 61.76 (75–120)	0.48
Operative time (mins)^a^	97.44 ± 15.33 (65–130)	96.73 ± 15.03 (60–250)	0.86

*PF* patellofemoal joint

^a^Values are expressed as mean ± SD, with range in parentheses

We have not seen any complications such as patellar crepitation, patellar fractures, infections, dislocation of the mobile polyethylene, lateral compartment arthritis, and component loosening in our study.

## Discussion

The patients who had progressive arthritis of the patellofemoral compartment and needed a revision TKA following UKA was minimal. Parrette et al. has shown that the mode of failure of a fixed bearing UKA in long-term follow up was from polyethylene wear, they found that only one patient had symptomatic patellofemoral arthritis and needed a revision TKA (Parratte et al. 2009). Foran et al. have also shown that the incidence of patellofemoral arthritis following a fixed bearing UKA were increased after long-term follow up. However, most patients did not have symptoms (Foran et al. 2013). Presently, the UKA procedure for patients who have medial OA knee with arthritis of the lateral facet of the patella remains a topic of debate among orthopedic surgeons. Few recent studies have shown good clinical results for this subgroup (Kang et al. 2011; Song et al. 2016). There is a belief that the reasons for reducing anterior knee pain should come first and that the cartilage damage and full thickness cartilage loss has been generally found in the elderly (Emery and Meachim 1973). Second, the overload to the patellofemoral compartment is reduced by the correction of any deformity to a normal alignment and generates a new contact surface between the femoral condyle and the patella (Beard et al. 2007a, b). Third, no impingement between the femoral condyle and the facet of the patella as the osteophyte at the femoral condyle and patella are removed during the surgery (Beard et al. 2007a, b). Therefore, patients with OA that have arthritis of the lateral facet of the patella would not be a contraindication to a UKA. This study aims to compare the results between patients with non-severe arthritis (group I) and severe arthritis (group II) of the lateral facet of the patella following a medial Oxford UKA. The post-operative outcomes were evaluated by assessing the incidence of anterior knee pain, the VAS for anterior knee pain, pain scores, functional scores, and knee scores.

This study has shown that only the knee scores of the group II patients were significantly worse than those in the group I patients. However, the other parameters including the incidence of post-operative anterior knee pain, VAS for post-operative anterior knee pain, post-operative pain scores, and post-operative functional scores were not significantly different between the groups.

The previous studies have shown similar results with our study. Song et al. used a fixed bearing UKA, and they selected the patients who had only mild to moderate arthritis of the medial and lateral facet of the patella seen in intra-operative findings. They have shown that the post-operative VAS for anterior knee pain of patients with patellar arthritis was low (1.7–2.3 points). Kang et al. have also shown that the patients with patellofemoral arthritis at medial and lateral facets on preoperative radiographies had shown good results following a medial Oxford UKA. Beard et al. have shown that preoperative anterior knee pain and intra-operative full thickness cartilage loss of the medial and lateral facet of the patella were not contraindications for the Oxford UKA. However, all of the previous studies did not include patients with ebernation of the lateral facet of the patella. In contrast, our study had 3 patients who presented with ebernation. Two patients had not experienced anterior knee pain following the surgery. Another one had anterior knee pain, but described it as only an occasional pain, mild in intensity.

Our study has also shown that the pain scores and functional scores were the same between the two groups but the knee society scores of patients in group II were worse than the patients in group I and this has shown a statistical significance. The reasons for lower knee society scores in group II patients in our study resulted from lower post-operative ROM and lower tibiofemoral angles. However, the post-operative pain scores and functional scores were similar. Therefore the group II patients also had good clinical outcomes that were not different from the group I patients. Bread et al. has also shown the similar results to those found in our study. They found that the Oxford knee score of patients with full-thickness cartilage loss of lateral facet of the patella had lower scores than the patients without. However these patients have shown good clinical outcomes. Kang et al. has shown that the Oxford knee score, SF-12 in patients with arthritis of the lateral facet and both patellar facets in preoperative radiographic results were the same as seen in patients without preoperative radiographic PF arthritis.

There are some limitations in our study. First, the subjects in our study were not randomized into the two groups as the finding of arthritis of the lateral facet of the patella would have been evaluated during surgery and thus the patients were classified into one or another group. However we were blinded in the evaluation of the anterior knee pain, pain scores and functional scores of the patients, post operatively. Second, the preoperative demographic data was not the same between the two groups. The patients with arthritis of the lateral facet of the patella have shown a lower ROM, lower knee scores, and more varus deformity than had been seen in patients without arthritis of the lateral facet of the patella. Third, we decided to determine the arthritis of the lateral facet of the patella through direct observation during the surgical procedure. There is a study from Koike et al. that had demonstrated that determining patellofemoral arthritis by a skyline view radiograph has shown to be inaccurate and it was technically demanding (Koike et al. 2015). Fourth, this study was done from a short follow-up. However our study did not find the progressive patellofemoral arthritis in both groups of patients following an Oxford UKA.

In conclusion, the medial OA knee patient with or without severe arthritis of the lateral facet of the patella has shown good clinical outcomes following a medial Oxford UKA.
